# A synergistic interaction between transcription factors nuclear factor-κB and signal transducers and activators of transcription 3 promotes gastric cancer cell migration and invasion

**DOI:** 10.1186/1471-230X-13-29

**Published:** 2013-02-12

**Authors:** Jiyeon Yoon, Sung Jin Cho, Young San Ko, Jinju Park, Dong Hoon Shin, In Chan Hwang, Sang Yeun Han, Seon Young Nam, Min A Kim, Mee Soo Chang, Hye Seung Lee, Woo Ho Kim, Byung Lan Lee

**Affiliations:** 1 Department of Anatomy, Seoul National University College of Medicine, Seoul, 110-799, South Korea; 2 Cancer Research Institute, Department of Tumor Biology, Seoul National University College of Medicine, Seoul, 110-799, South Korea; 3 Department of Economics, Cornell University, Ithaca, NY, 14853, USA; 4 Radiation Health Research Institute, Korea Hydro & Nuclear Power Co., Ltd, Seoul, 132-703, South Korea; 5 Department of Pathology, Seoul National University College of Medicine, Seoul, 110-799, South Korea; 6 Department of Pathology, Seoul National University Bundang Hospital, Seongnam, 463-707, South Korea; 7 Ischemic/Hypoxic Disease Institute Medical Research Center, Seoul National University College of Medicine, Seoul, 110-799, South Korea

## Abstract

**Background:**

The transcription factor nuclear factor-κB (NF-κB) has been implicated in gastric cancer metastasis, but the underlying molecular mechanisms remain unclear. We investigated the role of the interaction between NF-κB and signal transducers and activators of transcription 3 (STAT3) in controlling metastatic potential of gastric cancer cells.

**Methods:**

Immunohistochemistry for NF-κB p65 (RelA), phospho-Tyr705-STAT3 (pSTAT3), or matrix metalloproteinase 9 (MMP9) was performed on tissue array slides containing 255 gastric carcinoma specimens. NF-κB inhibition in SNU-638 and MKN1 gastric cancer cell lines were performed by transduction with a retroviral vector containing NF-κB repressor mutant of IκBα, and STAT3 was silenced by RNA interference. We also did luciferase reporter assay, double immunofluorescence staining and immunoblotting. Cell migration and invasion were determined by wound-healing assay and invasion assay, respectively.

**Results:**

NF-κB and STAT3 were constitutively activated and were positively correlated (*P* = 0.038) in gastric cancer tissue specimens. In cell culture experiments, NF-κB inhibition reduced STAT3 expression and activation, whereas STAT3 silencing did not affect NF-κB activation. Moreover, both NF-κB inhibition and STAT3 silencing decreased gastric cancer cell migration and invasion in a synergistic manner. In addition, both NF-κB activation and STAT3 activation were positively correlated with MMP9 in gastric cancer tissues (*P* = 0.001 and *P* = 0.022, respectively), decreased E-cadherin expression and increased Snail and MMP9 expressions in cultured cells.

**Conclusion:**

NF-κB and STAT3 are positively associated and synergistically contribute to the metastatic potential of gastric cancer cells. Thus, dual use of NF-κB and STAT3 inhibitors may enhance the efficacy of the anti-metastatic treatment of gastric cancer.

## Background

Metastasis is the major obstacle in the treatment of malignant cancer and it accounts for more than 90% of cancer-related mortality [1]. Since gastric cancer is the second most lethal cancer worldwide [2], the underlying molecular mechanisms responsible for gastric cancer metastasis need to be elucidated.

Nuclear factor-κB (NF-κB) is a family of signal-responsive transcription factors that includes RelA/p65, RelB, c-Rel, NF-κB1/p50 and NF-κB2/p52 [3]. NF-κB exists in an inactive form in the cytoplasm because of its interaction with the inhibitory protein, IκBα [4]. After activation of IκB kinases (IKKs), IκBs become phosphorylated, ubiquitinated and degradaded by proteasomes, which allows NF-κB to translocate to the nucleus, where it can activate or repress target genes [5]. With respect to gastric cancer, NF-κB is one of the most well-studied transcription factors, and is known to be activated by various factors, including cytokines [6-8], growth factors [9], Toll-like receptor signaling [10] and many other pathways of microbial recognition [11,12]. NF-κB activation has been frequently observed in both gastric cancer cells [13-16] and tumor-infiltrating lymphocytes [17]. In addition, down-regulation of NF-κB has been shown to suppress cell migration and invasion in gastric cancer cells *in vitro*[16,18]. Thus, modulation of the NF-κB pathway might be a promising therapeutic target for gastric cancer metastasis. However, the downstream mediators of NF-κB-induced metastasis in gastric cancer cells remain unclear.

Signal transducers and activators of transcription 3 (STAT3) belongs to the STAT family of signal-responsive transcription factors [19]. The inactive form of STAT3 in the cytoplasm of non-stimulated cells is activated by cytokines, growth factors and oncogenic proteins through sequential phosphorylation of tyrosine 705 and serine 727 [20]. Like NF-κB, constitutive activation of STAT3 has been shown to contribute to the progression of gastric cancer, including proliferation, apoptosis, angiogenesis and metastasis [21-23]. However, STAT3 showed differential roles in gastric cancer cell metastasis depending on the upstream regulator of STAT3 activation [21].

Previously, NF-κB activation in human cancers has been reported to be positively or negatively correlated with STAT3 activation in the control of tumorigenesis [24], tumor growth [3,24,25] and angiogenesis [26]. STAT3 activation increased NF-κB activation and tumor growth derived from cervical cancer cells or glioblastoma cells [25]. STAT3 also maintains NF-κB activation and retention in the nucleus in melanoma cells and prostate cancer cells [27]. In addition, NF-κB activation increased STAT3 activation through up-regulation of interleukin-6 (IL-6) in melanoma cells [26]. On the other hand, a positive crosstalk between NF-κB and STAT3 was found in B cell lymphoma, which increased cell proliferation and decreased apoptosis [3]. On the contrary, an inverse relationship between NF-κB and STAT3 was also shown in human hepatocellular carcinoma cells, in which IKKβ deletion increased STAT3 activation, tumorigenesis and tumor growth [24]. Thus, these findings indicate that the association between NF-κB and STAT3 could be different according to the cancer cell type investigated and, thus, interaction of these two molecules in terms of cancer cell metastasis in each cancer type needs to be elucidated.

Since the relationship between NF-κB and STAT3 pathways in gastric cancer has not been described previously, the present study performed a large scale immunohistochemical analysis to investigate the correlation between NF-κB p65 (RelA) and phospho-Tyr705-STAT3 (pSTAT3) or matrix metalloproteinase 9 (MMP9) in 255 surgically excised human gastric carcinoma tissues. In addition, we inhibited NF-κB in gastric cancer cells by transduction with a retroviral vector containing supersuppressive mutant form of IκBα (IκBαM) and silenced STAT3 by transfection of STAT3 small interfering RNA (siRNA). Then, we evaluated the effect of NF-κB and STAT3, alone or in combination, on the gastric cancer cell migration and invasion *in vitro*.

## Methods

### Patients and tissue array methods

A total of 255 surgically resected human gastric cancer specimens were obtained from the Department of Pathology, Seoul National University College of Medicine from January 1st to June 30th, 1995 and six paraffin array blocks were prepared by Superbiochips Laboratories (Seoul, Korea), as previously described [28]. Briefly, core tissue biopsies (2 mm in diameter) were taken from individual paraffin-embedded gastric tumors (donor blocks) and arranged in a new recipient paraffin block (tissue array block) using a trephine apparatus. The staining results of the different intratumoral areas of gastric carcinomas in these tissue array blocks showed an excellent agreement. A core was chosen from each case for analysis. We defined an adequate case as a tumor occupying more than 10% of the core area. Each block contained internal controls consisting of non-neoplastic gastric mucosa from body, antrum and other areas showing intestinal metaplasia. Sections of 4 μm thickness were cut from each tissue array block, deparaffinized, and rehydrated. This protocol was reviewed and approved by the Institutional Review Board of Seoul National University (Approval No. C-1006-035-320).

### Immunohistochemistry

Immunohistochemical staining was performed as described previously using a streptavidin peroxidase procedure (avidin-biotin complex method) after antigen retrieval using an autoclave [29]. The primary antibodies used were anti-NF-κB/RelA (1:50, Santa Cruz Biotechnology, Santa Cruz, CA, USA), anti-phospho-Tyr705-STAT3 (1:50, Cell Signaling Technology, Beverly, MA, USA), anti-MMP9 (1:150, Neomarkers, Fremont, CA, USA). Immunostaining results were considered to be positive if ≥ 10% (for nuclear NF-κB/RelA and cytoplasmic MMP9) or ≥ 5% (for nuclear pSTAT3) of the neoplastic cells were stained.

### Cell culture

SNU-638 and MKN1, which are well characterized human gastric cancer cell lines [30-32], were purchased from the Korean Cell Line Bank (Seoul, Korea). Cells were cultured in RPMI1640 (Life Technologies, Grand Island, NY, USA) supplemented with 10% fetal bovine serum (FBS), 2 mg/mL sodium bicarbonate, 100 U/mL penicillin, and 100 μg/mL streptomycin (Life Technologies) at 37°C in a humidified 95% air and 5% CO_2_ atmosphere.

### Infection with retroviral vectors expressing IκBα supersuppressor

The control retroviral vector MFG.EGFP.IRES.puro has been previously described [33]. MFG.EGFP.IRES.puro and the retroviral vector MFG.IκBα.IRES.puro, which encodes a supersuppressive mutant form of IκBαM, were generated and infected into gastric cancer cells, as described previously [34]. Pooled puromycin-resistant cells were used for further analysis.

### STAT3 siRNA transfection

STAT3 siRNA and scrambled siRNA (control) were purchased from Santa Cruz Biotechnology. STAT3 siRNA or control siRNA was then transfected into gastric cancer cells using LipofectAMINE Plus (Life Technologies) according to the manufacturer’s instructions.

### Preparation of nuclear and cytoplasmic extracts

Cells were scraped and lysed in cold lysis buffer A [10 mM Tris (pH 8.0), 60 mM NaCl, 1 mM EDTA, 1 mM DTT, 0.1% Nonidet P-40 (NP-40), 1 mM phenylmethylsulfonyl fluoride], incubated on ice for 10 min, centrifuged, and the cytoplasmic extracts obtained were transferred to fresh tubes. For nuclear extracts, the pelleted nuclei were washed in 1 mL buffer A without NP-40 and resuspended in 50 μL cold lysis buffer B [200 mM HEPES (pH 7.9), 0.75 mM spermidine, 0.15 mM spermine, 0.2 mM EDTA, 2 mM EGTA, 2 mM DTT, 20% glycerol, 1 mM phenylmethylsulfonyl fluoride, 0.4 M NaCl]. They were then extracted on ice for 10 min with occasional vortexing. The lysate was centrifuged at 170 g at 48°C for 2 min, and the supernatant was collected as nuclear extracts.

### Immunoblotting

Cell lysates were prepared in 100–200 μL of 1x sodium dodecyl sulfate (SDS) lysis buffer [125 mM Tris–HCl (pH 6.8), 4% SDS, 0.004% bromophenol blue, and 20% glycerol]. Protein contents were measured using BCA Protein Assay Reagent (Pierce, Rockford, IL, USA). Equal amounts of proteins were loaded onto a 10% discontinuous SDS/polyacrylamide gel and electrophoretically transferred to PVDF membranes (Millipore Corporation, Billerica, MA, USA) blocked with 5% non-fat dry milk in phosphate-buffered saline-Tween-20 (0.1%, v/v) for 1 h. The membranes were then incubated at 4°C overnight with or without 2 h incubation at room temperature with one of the following primary antibodies: anti-RelA (1 : 1000, Santa Cruz Biotechnology), anti-phospho-Ser536-RelA (pRelA; 1:1000; Cell Signaling Technology), anti-STAT3 (1:500, Santa Cruz Biotechnology), anti-phospho-Tyr705-STAT3 (1:1000, Cell signaling Technology), anti-E-cadherin (1:1000, BD Biosciences, San Jose, CA, USA), anti-Snail (1:1000, Santa Cruz Biotechnology), anti-MMP9 (1:1000, Neomarkers), anti-β-actin (1:1000, Sigma, St Louis, MO, USA) and anti-TFIIB (1:1,000, BD Bioscience). Horseradish peroxidase-conjugated anti-rabbit IgG (1:2000, Zymed, San Francisco, CA, USA) or anti-mouse IgG (1:2500, Santa Cruz Biotechnology) was used as a secondary antibody. Enhanced chemiluminescence was used to detect the immunoreactive proteins. Equal protein loading was confirmed by β-actin (whole-lysate protein) or TFIIB (nuclear protein).

### Transient transfection and luciferase reporter assay

The NF-κB-luciferase reporter plasmid (pNF-κB-luciferase) (Stratagene, La Jolla, CA, USA) contains a 5x NF-κB response element fused to luciferase. The STAT-luciferase reporter plasmid (pSTAT-luciferase) (Addgene plasmid 8688) contains four copies of the sequence GGTTCCCGTAAATGCATCA (TTCCCGTAA is the STAT-binding site) fused to luciferase. SNU-638 cells were transiently co-transfected with 0.4 μg of luciferase reporter plasmid and 0.4 μg of β-galactosidase vector, an internal control, using LipofectAMINE Plus (Life Technologies). Twenty-four hours after transfection, assays for luciferase and β-galactosidase were carried out using a Dual-Luciferase Reporter Assay System (Promega, Madison, WI, USA). Luciferase activity was measured on an AutoLumat LB 9505c luminometer (Berthold Analytical Instruments, Nashua, Germany) and was normalized by β-galactosidase activity. Luciferase activity in control cells was arbitrarily set to 1.

### Immunofluorescence staining

Cells were cultured on chamber slides. After 24 h, cells were fixed with 4% paraformaldehyde, permeabilized with 0.5% Triton X-100 for 5 min, and blocked with 5% normal donkey serum. After blocking, cells were incubated overnight at 4°C with mixture of the following primary antibodies: anti-pRelA (1:200; Cell Signaling Technology) and anti-STAT3 (1:200, Santa Cruz Biotechnology). Alexa fluor-555-conjugated anti-rabbit IgG (1:200, Life Technologies) and −488-conjugated anti-mouse IgG (1:200, Life Technologies) were used as secondary antibodies. Cells were stained with 4^′^,6-diamidino-2-phenylindole (Life Technologies), which was used for nuclear staining. Immunofluorescence was detected with a fluorescence microscope.

### Wound-healing assay

Cells were cultured in 6-well plates until confluent. The cell monolayer was scratched with a sterile pipette tip to generate a wound. The remaining cells were washed twice with culture medium to remove cell debris. Spontaneous cellular migration was monitored using a phase contrast microscope and captured using an Olympus Digital Camera (Olympus, Tokyo, Japan) at 0, 24 and 48 h. The area of the scratches was measured and quantified using NIH Image Analysis software (version 1.62; National Institute of Health, Bethesda, MD, USA).

### Cell invasion assay

A 24-well Insert System using an 8 μm polyethylene terephthalate membrane was obtained (BD biosciences) and coated with Matrigel (BD Biosciences). Inserts were rehydrated with RPMI1640 for 2 h at room temperature prior to use. After rehydration, media was removed and cells (1 × 10^5^ cells/insert) were added to the top of each insert chamber in RPMI1640 containing 1% FBS. Lower chamber contained the medium with 10% FBS as a chemoattractant. After incubation for 48 h, non-invading cells were carefully removed from the top of each insert with a cotton swab. Invasive cells were stained with 0.2% crystal violet in 20% methanol as described previously [35] and were observed with an inverted microscope. Stained cells also dissolved in 10% SDS, and absorbance was measured at 570 nm using an ELISA reader (Bio-Rad, Hercules, CA, USA).

### Statistical analysis

For tissue array analysis, statistical analyses were conducted using SPSS version 11.0 statistical software program (SPSS, Chicago, IL, USA), and the chi-squared test was used to determine the correlations between the expressions of NF-κB, pSTAT3, and MMP9. For cell culture experiments, data were analyzed using GraphPad Prism software for Windows Vista (version 4; GraphPad Software, San Diego, CA, USA) and the two-tailed Student’s *t*-test was used to determine the significances of the results. *P* values of < 0.05 were considered statistically significant for all statistical analyses.

## Results

### NF-κB, pSTAT3 and MMP9 are positively correlated with each other in clinical gastric cancer specimens

**Figure 1:**
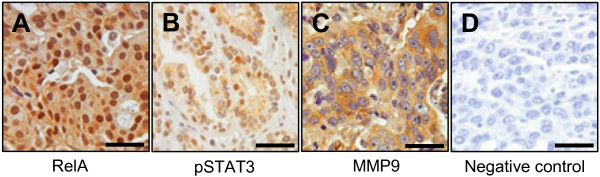
**Representative immunohistochemical features of NF-κB, pSTAT3 and MMP9 in human gastric cancer specimens.** (**A**) NF-κB RelA (p65)-positive. (**B**) pSTAT3-positive. (**C**) MMP9-positive. (**D**) Negative control treated without the primary antibodies. Scale bars = 100 μm.

Representative results of the immunohistochemical staining are shown in Figure 1. Immunoreactivity for NF-κB and pSTAT3 were found in both the nuclei and cytoplasm of tumor cells. Cells showing distinct nuclear staining, regardless of the presence of cytoplasmic staining, were considered to express activated forms of NF-κB (Figure 1A) or STAT3 (Figure 1B). On the other hand, the expression of MMP9 was detected mainly in the cytoplasm of tumor cells (Figure 1C). Positive immunoreactivity for nuclear NF-κB was found in 41 of 255 (16%) of clinical samples of gastric cancer. In addition, the expression of nuclear pSTAT3 and cytoplasmic MMP9 were found in 61 of 255 (24%) and 46 of 255 (18%) of gastric cancer specimens, respectively. Data concerning the correlations between NF-κB activation, STAT3 activation, and MMP9 expression are summarized Table 1. NF-κB activation was found to be significantly and positively correlated with STAT3 activation (*P* = 0.038) and MMP9 expression (*P* = 0.001). Similarly, STAT3 activation was also correlated with MMP9 expression (*P* = 0.022).

**Table 1:** Correlation between expressions of NF-κB, pSTAT3 and MMP9 in human gastric cancer specimens

	**NF-κB**		***P***
	**Positive (%)**	**Negative (%)**	
Total	41 (16)	214 (84)	
pSTAT3			
Positive	15 (25)	46 (75)	0.038*
Negative	26 (13)	168 (87)	
MMP9			
Positive	15 (33)	31 (67)	0.001*
Negative	26 (12)	183 (88)	
	pSTAT3		*P*
	Positive (%)	Negative (%)	
Total	61 (24)	194 (76)	
MMP9			
Positive	17 (37)	29 (63)	0.022*
Negative	44 (21)	165 (79)	

*NF-κB*, nuclear factor-κB; *pSTAT3*, phospho-Tyr705-signal transducers and activators of transcription 3; *MMP9*, matrix metalloproteinase 9.

* Considered to be statistically significant (< 0.05).

### IκBαM overexpression reduces STAT3 expression and activation

**Figure 2:**
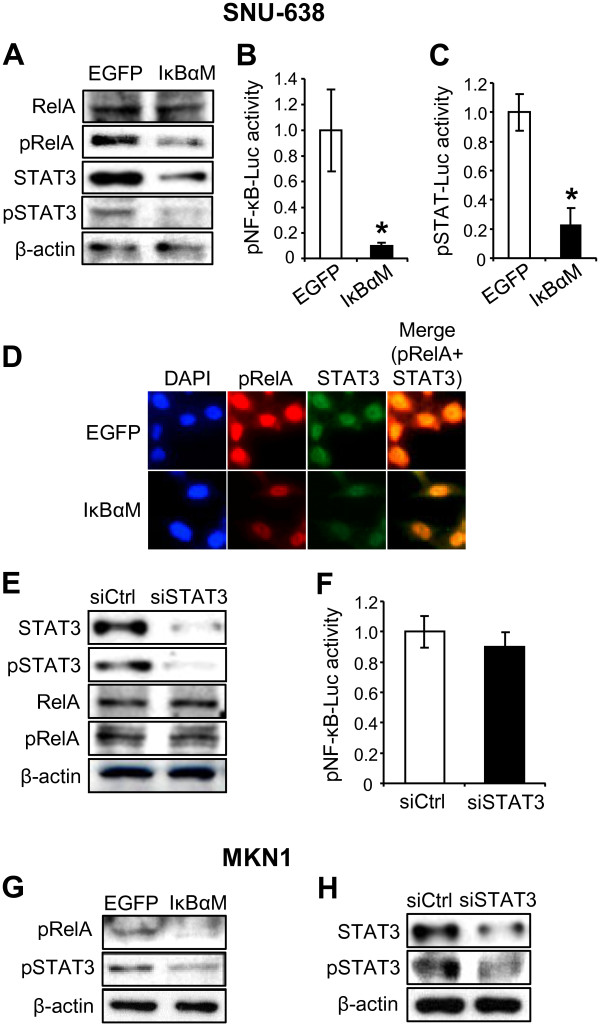
**Effect of downregulation of NF-κB p65 on the STAT3 activation in gastric cancer cells and vice versa.** (**A-D**) SNU-638 cells were infected with either MFG.IκBαM.IRES.puro (IκBαM) retroviral vector or empty (EGFP) vector. (**A**) The protein expressions of total NF-κB p65 (RelA), phospho-Ser536-NF-κB p65 (pRelA), total STAT3 and phosphor-Tyr705-STAT3 (pSTAT3) were determined by immunoblotting. (**B**) Cells were transiently co-transfected with pNF-κB-luciferase and a β-galactosidase vector, and effect of IκBαM overexpression on NF-κB transcriptional activity was determined by luciferase reporter assay. Each bar represents the mean ± SD (n = 6). * *P* < 0.05 versus control cells infected with an empty vector. (**C**) Cells were transiently co-transfected with pSTAT-luciferase and a β-galactosidase vector, and effect of IκBαM overexpression on STAT transcriptional activity was determined by luciferase reporter assay. Each bar represents the mean ± SD (n = 5). * *P* < 0.05 versus control cells infected with an empty vector. (**D**) 4’,6’-Diamidino-2-phenylindole staining (blue) and double immunofluorescence staining showing the nuclear colocalization of pRelA (red) and STAT3 (green). (**E** and **F**) SNU-638 cells were transiently transfected with control siRNA (siCtrl) or STAT3 siRNA (siSTAT3). (**E**) The effects of STAT3 silencing on the protein expressions of total STAT3, pSTAT3, total RelA and pRelA were determined by immunoblotting. (**F**) Luciferase reporter assay was performed to show the regulation of NF-κB activation by STAT3 after STAT3 silening. Each bar represents the mean ± SD (n = 6). * *P* < 0.05 versus control siRNA-transfected cells. (**G**) Immunoblotting for pRelA and pSTAT3 in MKN1 cells after IκBαM overexpression. (**H**) Immunoblotting for total STAT3 and pSTAT3 in MKN1 cells after STAT3 silencing.

Since the relationship between NF-κB and STAT3 has been dependent on the cellular context and cell type [3,24-27], we performed *in vitro* experiments. To investigate whether STAT3 is regulated by NF-κB, we produced stable cell lines from SNU-638 and MKN1 cells overexpressing IκBαM. Immunoblotting analysis (Figure 2A) was performed to determine the protein expression of NF-κB p65 subunit phosphorylated at serine-536 (pRelA) in addition to the protein expression of total NF-κB p65 (RelA), because an important site of phosphorylation of NF-κB p65 subunit is at serine-536, and this phosphorylation is involved in regulation of transcriptional activity, nuclear localization, and protein stability [36]. Our results showed that NF-κB activation (manifested as pRelA expression) was down-regulated, whereas total RelA protein expression was not modulated. Consistently, luciferase reporter assay also showed that NF-κB transcriptional activity markedly decreased in IκBαM-overexpressing cells (Figure 2B). Then, we assessed whether NF-κB regulates the STAT3 activation by immunoblotting and found that IκBαM overexpression decreased the STAT3 expression and activation (manifested as pSTAT3 expression) (Figure 2A and 2G). STAT luciferase reporter assay also showed that STAT transcriptional activity was decreased in IκBαM-overexpressing cells (Figure 2C). In addition, double immunofluorescence staining showed that pRelA and STAT3 were colocalized in the nucleus of the same gastric cancer cells, which was reduced in IκBαM-overexpressing cells (Figure 2D).

Next, to investigate whether there is a crosstalk between NF-κB and STAT3, STAT3 was silenced by transfection of STAT3 siRNA. Immunoblotting showed that STAT3 silencing decreased STAT3 expression and activation, but neither total RelA nor pRelA expression was changed in STAT3-silenced cells (Figure 2E and 2H). In addition, luciferase reporter assay confirmed that STAT3 silencing did not modulate NF-κB transcriptional activity (Figure 2F). Taken together, these findings suggest that STAT3 acts as a downstream molecule of NF-κB in NF-κB pathway.

### NF-κB suppression decreases the migration and invasion through the regulation of EMT markers

**Figure 3:**
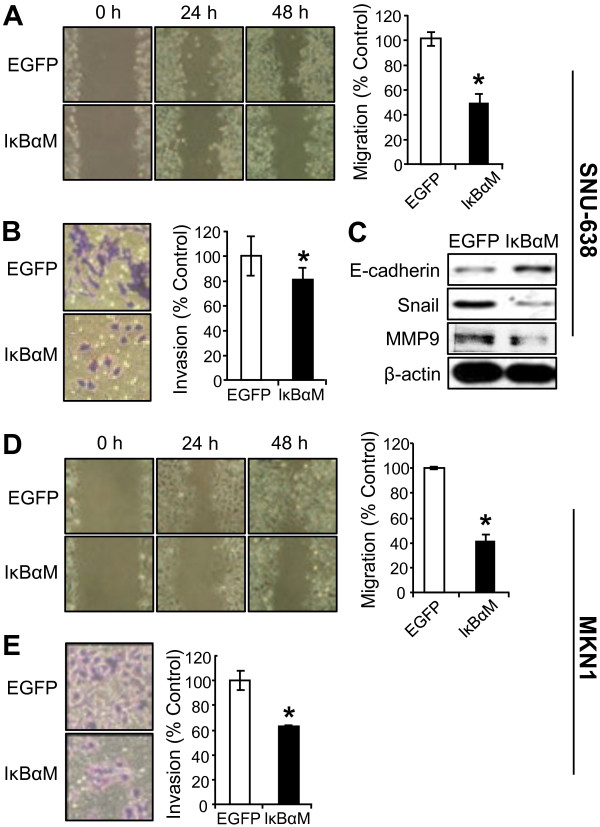
**Effect of IκBαM overexpression on metastatic potential of gastric cancer cells.** SNU-638 (**A-C**) cells and MKN1(**D** and **E**) cells were infected with either IκBαM retroviral vector or an empty (EGFP) vector. (**A** and **D**) Confluent cell monolayers were wounded and cell migration was analyzed by wound-healing assay 48 h after scratching. (**B** and **E**) Cells were seeded in the upper chamber coated with Matrigel and invasion ability was measured 48 h after cell plating. Results were calculated as percentages relative to control cells infected with an empty vector. Each bar represents the mean ± SD (n = 4). * *P* < 0.05 versus control cells infected with an empty vector. (**C**) The protein expressions of E-cadherin, Snail, MMP9 and β-actin expressions were determined by immunoblotting.

In the initial steps of metastasis of carcinoma cells, epithelial cancer cells change their phenotype to mesenchymal phenotype and become motile and invasive by a process called epithelial-mesenchymal transition (EMT) [37]. This process includes down-regulation of epithelial markers and up-regulation of mesenchymal markers [37]. To confirm the effect of NF-κB activation on gastric cancer cell motility, we used a stable SNU-638 and MKN1 cells overexpressing IκBαM. Wound-healing assay showed that IκBαM overexpression significantly decreased migration of gastric cancer cells (by 51% in SNU-638 cells and by 59% in MKN1 cells) compared with control cells infected with an empty vector (Figure 3A and 3D). Moreover, invasion assay also showed that IκBαM overexpression decreased invasion of gastric cancer cells (by 28% in SNU-638 cells and by 37% in MKN1 cells) compared with control cells (Figure 3B and 3E).

To further examine the role of NF-κB in EMT of gastric cancer cells, we analyzed the effect of NF-κB inhibition on the expressions of representative EMT marker proteins. Immunoblotting showed that the expression of E-cadherin, a representative epithelial marker, increased, whereas the expression of mesenchymal markers Snail and MMP9 decreased after IκBαM overexpression (Figure 3C).

### STAT3 silencing decreases the migration and invasion through regulation of EMT markers

**Figure 4:**
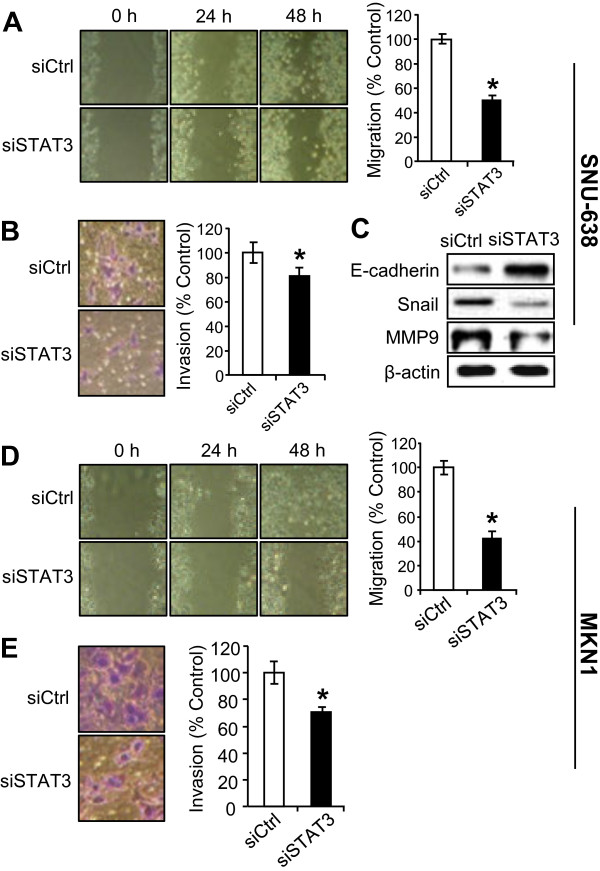
**Effect of STAT3 silencing on metastatic potential of gastric cancer cells.** SNU-638 cells (**A-C**) and MKN1 cells (**D** and **E**) were transiently transfected with control siRNA (siCtrl) or STAT3 siRNA (siSTAT3). (**A** and **D**) Cell migration capacity was evaluated by wound-healing assay 48 h after scratching. (B and E) Cell invasion ability was measured 48 h after cell plating. Results were calculated as percentages relative to control siRNA-transfected cells. * *P* < 0.05 versus control cells (n = 4). (**C**) The protein expressions of E-cadherin, Snail, MMP9 and β-actin in SNU-638 cells were determined by immunoblotting after STAT3 silencing.

Next, we confirmed the effects of STAT3 silencing on the motility and invasiveness in gastric cancer cells. As expected from the previous report [21], STAT3 silencing suppressed cell migration (by 49% in SNU-638 cells and by 57% in MKN1 cells) compared with control siRNA-transfected gastric cancer cells (Figure 4A and 4D). Moreover, STAT3 silencing also decreased invasiveness (by 27% in SNU-638 cells and by 30% in MKN1 cells) compared with control cells (Figure 4B and 4E). We found that E-cadherin increased, whereas Snail and MMP9 decreased after transfection of STAT3 siRNA (Figure 4C).

### NF-κB and STAT3 cooperatively induce migration and invasion of gastric cancer cells

Our results in the present study showed that NF-κB and STAT3 played important roles in migration and invasion, and that NF-κB was an upstream regulator of STAT3. To examine the combined effect of NF-κB and STAT3 on the metastatic potential of gastric cancer cells, we performed co-transfection of IκBαM and STAT3 siRNA into SNU-638 cells.

**Figure 5:**
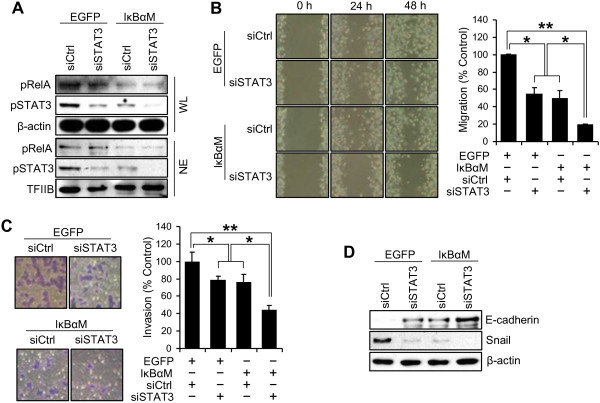
**Synergistic effect of NF-κB and STAT3 on metastatic potential of gastric cancer cells.** Stable SNU-638 cells infected with either IκBαM retroviral vector or an empty (EGFP) vector were transiently co-transfected with either control siRNA (siCtrl) or STAT3 siRNA (siSTAT3). (**A**) Whole cell lysates (WL) and nuclear extracts (NE) were obtained 24 h after co-transfection and protein levels for pRelA, pSTAT3 and β-actin or TFIIB were measured by immunoblotting. (**B**) Effect of interaction of NF-κB and STAT3 on cell migration capacity was evaluated by wound-healing assay 48 h after scratching. (**C**) Effect of interaction of NF-κB and STAT3 on cell invasion ability was measured by invasion assay 48 h after cell plating. Results were calculated as percentages relative to controls. Each bar represents the mean ± SD (n = 4). * *P* < 0.05 and ** *P* < 0.005. (**D**) The protein expressions of E-cadherin, Snail and β-actin in SNU-638 cells were determined by immunoblotting after co-transfection of IκBαM and siSTAT3.

To confirm the effects of co-transfection of IκBαM and STAT3 siRNA on expression of pRelA and pSTAT3, we obtained whole cell lysates and nuclear extracts and performed immunoblotting. We found that double knock-down of RelA and STAT3 induced marked down-regulation of pSTAT3 expression in both the whole cell lysates (1st–3rd rows in Figure 5A) and nuclear extracts (4th–6th rows in Figure 5A).

In quantitative terms, the migration capacity decreased by 50% in IκBαM-overexpressing cells, and by 45% in STAT3-slienced cells compared with control cells. In the co-transfected cells, the migration capacity was remarkably inhibited when STAT3 was further silenced (81% lower than that of the control) (Figure 5B). Similarly, invasion assay showed that cells with down-regulation of both NF-κB and STAT3 showed lower invasion ability (56% lower than that of the control) than those with down-regulation of either alone (Figure 5C). These data suggest that STAT3 in this system is induced not only through NF-κB, but also through something else. It is known that STAT3 pathway can be induced by many NF-κB independent pathways including some cytokines [38] and tyrosine kinases [39]. We also found that E-cadherin expression was increased whereas Snail expression was decreased in cells with down-regulation of both NF-κB and STAT3 compared with those with down-regulation of either alone (Figure 5D).

## Discussion

Understanding of a clear regulatory path of signaling molecules in cancer cells is a pre-requisition to successful co-development of therapeutic targets for tumors. Since the pivotal role of NF-ĸB in gastric cancer progression has been shown [16,18], a thorough understanding of NF-ĸB pathway can lead to future studies and drug development which could provide a novel option in the treatment of this disease. Although both NF-κB and STAT3 are shown to be involved in the metastasis of gastric cancer [16,18,21], the link between NF-ĸB and STAT3 has not been validated. In the present study, we investigated the relationship between NF-κB and STAT3 in terms of gastric cancer metastasis. To the best of our knowledge, this is the first study to show the association between NF-κB and STAT3 in gastric cancer.

In the present study, constitutive activation of NF-κB and STAT3 was found in 16% and 24% of 255 gastric cancer specimens, respectively, and they showed a positive correlation (*P* = 0.038). In addition, our *in vitro* experiments showed that NF-κB inhibition reduced the protein expression of total STAT3 and pSTAT3, which was possibly caused by the suppression of STAT3 at the transcriptional or translational level. Since we wondered whether there is a reciprocal regulatory loop between NF-κB and STAT3, we further analyzed the effect of STAT3 silencing on the NF-κB activation. However, we found that STAT3 did not affect either NF-κB expression or activation. Thus, these results suggest that STAT3 is a downstream molecule of NF-κB in NF-κB pathway. Our observations contrast with a report by Yang *et al.* (2007), which showed that STAT3 and RelA can heterodimerize to transcriptionally regulate NF-κB-dependent genes [40]. Although Wani *et al.* (2011) reported that NF-κB activation induced STAT3 activation mediated by IL-6 [26], the present study did not show whether IL-6 is reduced in the SNU-638 cells overexpressing IκBαM, which may account for the reduced STAT3 levels. Thus, further investigations are needed to obtain a better understanding of the mechanism involved in NF-κB-induced STAT3 activation.

EMT confers acquisition of cell migration and invasion as well as molecular alterations in cancer cells [37]. Although the existence of EMT has not been shown in all types of cancers, previous studies have demonstrated that EMT plays a key role in the malignant progression of gastric cancer by using gastric cancer cell lines, orthotopic xenograft tumors and surgical gastric cancer specimens [41-43]. In the present study, we showed that IκBαM overexpression decreased the migration and invasion of gastric cancer cells. Moreover, IκBαM overepxression increased E-cadherin expression and decreased Snail expression, which indicates the change toward the mesenchymal phenotype. Thus, these results indicate that NF-κB might contribute to malignant progression through promotion of EMT.

Regarding the role of STAT3 in gastric cancer cells, Okamoto *et al.* (2011) found that STAT3 activation induced cancer cell motility through the Janus kinase pathway, whereas it enhanced survival of MET-activated gastric cancer cells [21]. Thus, they concluded that STAT3 plays differential roles depending on the upstream regulator of STAT3 activation in gastric cancer cells. In the present study, STAT3 silencing decreased the migration and invasion in SNU-638 gastric cancer cells with high NF-κB activity [44]. These findings, thus, suggest that STAT3 activation through NF-κB could contribute to the metastatic potential of a subset of gastric cancer cells.

MMP9 plays a critical role in maintaining the degradation and synthesis of extracellular matrix, and was shown to be positively associated with gastric cancer cell metastasis in animal models [45] and human gastric cancers [46]. Here, we examined the MMP9 expression and found that both NF-κB and STAT3 activation were positively correlated with MMP9 expression in clinical gastric cancer samples and in cultured cells. However, Table 1 showed that there are much more MMP9-positive cells (18%) than cells with activation of both NF-κB and STAT3 (6%). Therefore, we speculate that MMP9 can be induced by many other pathways independent on NF-κB/STAT3 signaling pathway in gastric cancer [47,48].

Although targeted therapies may offer enhanced efficacy and improved selectivity (and therefore less toxicity), mostly their effects are not durable when they are used alone. For this reason, combination therapies are often needed to effectively treat many tumors [49]. In the present study, we found that the combination of NF-κB inhibition and STAT3 silencing further reduced migration and invasion of gastric cancer cells compared to down-regulation of each molecule. Therefore, NF-κB and STAT3 seems to act in a synergistic manner in modulating migration and invasion of gastric cancer cells.

## Conclusions

Our results suggest that NF-κB and its downstream molecule STAT3 synergistically promote the metastatic potential of gastric cancer cells. Thus, the targeted combination therapy using NF-κB and STAT3 inhibitors appears to be a good approach to combat gastric cancer metastasis.

## Abbreviations

NF-κB: Nuclear factor-κB; STAT3: Signal transducers and activators of transcription 3; pSTAT3: Phospho-Tyr705-STAT3; MMP9: Matrix metalloproteinase 9; siRNA: Small interfering RNA; FBS: Fetal bovine serum; SDS: Sodium dodecyl sulfate; pRelA: NF-κB p65 subunit phosphorylated at serine-536; EMT: Epithelial-mesenchymal transition.

## Competing interests

The authors declare that they have no competing interests.

## Authors’ contributions

JY and SJC have made substantial contributions to acquisition of data, and analysis and interpretation of data, as well as have been involved in drafting the manuscript. YSK, JP, DHS, ICH and SYH have made substantial contributions to acquisition of data, and analysis of data. MAK, MSC, HSL, SYN and WHK have made substantial contributions to analysis and interpretation of data. All authors have given final approval of the version to be published. BLL has made substantial contributions to conception, design and drafting the manuscript and revising it critically for important intellectual content.

## Pre-publication history

The pre-publication history for this paper can be accessed here:

http://www.biomedcentral.com/1471-230X/13/29/prepub
